# Activation of TREK currents by riluzole in three subgroups of cultured mouse nodose ganglion neurons

**DOI:** 10.1371/journal.pone.0199282

**Published:** 2018-06-21

**Authors:** Diego Fernández-Fernández, Alba Cadaveira-Mosquera, Lola Rueda-Ruzafa, Salvador Herrera-Pérez, Emma L. Veale, Antonio Reboreda, Alistair Mathie, J. Antonio Lamas

**Affiliations:** 1 Department of Functional Biology and Health Sciences, Faculty of Biology–CINBIO, University of Vigo, Vigo, Galicia, Spain; 2 Medway School of Pharmacy, University of Kent, Chatham Maritime, Kent, United Kingdom; Indiana University School of Medicine, UNITED STATES

## Abstract

Two-pore domain potassium channels (K2P) constitute major candidates for the regulation of background potassium currents in mammalian cells. Channels of the TREK subfamily are also well positioned to play an important role in sensory transduction due to their sensitivity to a large number of physiological and physical stimuli (pH, mechanical, temperature). Following our previous report describing the molecular expression of different K2P channels in the vagal sensory system, here we confirm that TREK channels are functionally expressed in neurons from the mouse nodose ganglion (mNG). Neurons were subdivided into three groups (A, Ah and C) based on their response to tetrodotoxin and capsaicin. Application of the TREK subfamily activator riluzole to isolated mNG neurons evoked a concentration-dependent outward current in the majority of cells from all the three subtypes studied. Riluzole increased membrane conductance and hyperpolarized the membrane potential by approximately 10 mV when applied to resting neurons. The resting potential was similar in all three groups, but C cells were clearly less excitable and showed smaller hyperpolarization-activated currents at -100 mV and smaller sustained currents at -30 mV. Our results indicate that the TREK subfamily of K2P channels might play an important role in the maintenance of the resting membrane potential in sensory neurons of the autonomic nervous system, suggesting its participation in the modulation of vagal reflexes.

## Introduction

Mammalian two-pore-domain potassium (K2P) channels, discovered in 1996 [1], have been shown to be expressed in many neuronal types of the central and peripheral somatic nervous system [2–4]. However, only a small group of pioneering studies have reported that K2P channels are also expressed in neurons of the rat and mouse autonomic nervous system [5–7]. Although there are few data on the membrane properties of mouse nodose ganglion (mNG) neurons, we have previously reported expression of the TREK-1 subtype of K2P channels in these neurons using molecular techniques and single-channel recording. When riluzole, a well-known activator of TREK channels, was applied to these neurons, an outward current was observed [6]. The nodose ganglion is a complex structure containing several neuronal types with different electrical properties, and innervating several internal organs [8]. As it is currently unknown whether all neuronal subtypes express TREK channels, we have investigated the expression profile of this K2P subfamily. It was therefore important initially to choose an appropriate strategy to classify the mNG neurons isolated *in vitro*.

In the somatosensory system, afferent fibers arising from the dorsal root ganglia (DRG) are traditionally classified into four categories. This is mainly based on the conduction velocity (CV) of their axons, where the faster rat DRG fibers are referred to as type Aα (30–55 m.s^-1^) and Aβ (14–30 m.s^-1^), and the slower fibers as type Aδ (2.2–8 m.s^-1^) and C (<1.4 m.s^-1^) [9, 10]. The categorization is also defined by the degree of myelination, where C fibers are unmyelinated and A fibers strongly myelinated.

In the same way as in the DRG, attempts have been made to obtain a comparable taxonomy for sensory vagal afferents. Li and Schild (2007) classified rat NG (rNG) neurons into three subtypes depending on CV obtained in slices: A (10–18 m.s^-1^), Ah (4–18 m.s^-1^) and C (< 1 m.s^-1^) [11]. They proposed a further classification of isolated neurons based on the action potential wave-shape analysis and their complementary sensitivity to selective vanilloid and purinergic receptor agonists (capsaicin and ATP). This would allow discernment among these categories even when neurons are cultured in isolation, and thus CV cannot be estimated. Similarly, Kollarik et al. (2003) distinguished four groups of fibers in the mouse vagus nerve: two types of fast A-fibers, the fastest conducting at more than 15 m.s^-1^ and the other at about 6 m.s^-1^; and two types of slower C-fibers conducting at 0.9 and 0.5 m.s^-1^. Interestingly, only the slowest C-fibers were reported to be sensitive to capsaicin [12].

On this basis, we classified the mNG neurons into three groups based on their response to capsaicin and tetrodotoxin (TTX). Cells unresponsive to capsaicin and without TTX resistant sodium currents were classified as A-type, those unresponsive to capsaicin and showing both TTX-sensitive and resistant (TTX-SR) sodium currents were Ah-type, and cells responding to capsaicin and having TTX-SR currents were defined as C-type.

Using this newly developed classification of isolated mNG neurons, we report here that all types of nodose ganglion neurons respond to riluzole and other TREK modulators, suggesting that they express one or more types of TREK subfamily channels (TREK-1, TREK-2 and TRAAK). Thus, we propose that TREK channels might participate and be important not only in the regulation of several vagal reflexes, but also in the visceral unconscious sensory detection as their activity is modulated by several physiological stimuli such as temperature, mechanical deformation and pH changes.

## Material and methods

Swiss CD1 mice were obtained from the animal facility of The Biomedical Research Centre of the University of Vigo. Animals were grouped in standard cages, kept in a 12 h/12h light/dark cycle with food and water *ad libitum*. They were maintained and handled in accordance with the experimental procedures approved by the Spanish Research Council and the University of Vigo Scientific Committee. They observed the Spanish and European directives for the protection of experimental animals (RD1201/2005; 86/609/EEC).

### Nodose ganglion primary cell culture

Adult nodose ganglion neuron cultures were prepared from 30–60 days old mice (N = 52) as previously described for superior cervical ganglion [13, 14]. Briefly, mice were anaesthetized in a carbon dioxide (CO_2_) chamber and sacrificed by decapitation. Both nodose ganglia were identified from the ventral neck, quickly isolated, cleaned and subsequently incubated with collagenase (15 minutes, 2.5 mg.mL^-1^ in Hanks’ Balanced Salt Solution, HBSS) and trypsin (30 minutes, 1 mg.mL^-1^ in HBSS), and gently dissociated with a Pasteur pipette. After centrifugation (500 rpm, 3 min), the pellet was resuspended in 600 μL of L-15 modified medium supplemented with 10% foetal calf serum, 24 mM NaHCO_3_, 38 mM D-glucose, 100 UI.mL^-1^ penicillin-100 μg.mL^-1^ streptomycin, 2 mM L-glutamine and 50 ng.mL^-1^ nerve growth factor. The dissociated neurons were then plated onto laminin-coated (10 mg.mL^-1^ in Earle’s Balanced Salts Solution, EBSS) 35 mm dishes (200 μL suspension per dish) and kept in an incubator gassed with 95% oxygen (O_2_)-5% CO_2_ at 37°C for at least 24 hours, until recordings were made. All solutions were purchased from Sigma-Aldrich (Madrid, Spain). This protocol has been described in detail and is electronically available in Protocols.io: (http://dx.doi.org/10.17504/protocols.io.kzxcx7n [PROTOCOL DOI]).

### Recordings from nodose ganglion neurons

For patch-clamp experiments, cells with minimal neuronal processes were selected in order to avoid space-clamp associated errors [13]. The culture was maintained under continuous perfusion (~10 mL.min^-1^) at room temperature. An Axopatch 200B (Axon Instruments, Foster City, USA) amplifier was used to perform the experiments. Two-step fire-polished pipettes (4–6 MΩ of tip resistance) were pulled to have final access resistances below 20 MΩ and filled with pipette solution containing amphoterincin-B (75 μg.mL^-1^).

Current-clamp experiments were performed with the “Iclamp-fast” mode (simulating bridge mode) [15] in extracellular solution containing (in mM): NaCl 140, KCl 3, MgCl_2_ 1, CaCl_2_ 2, D-glucose 10, HEPES 10, gassed with O_2_; pH of 7.2 was adjusted with Tris (Tris(hydroxymethyl)-amino methane). Pipette solution contained (in mM): K-acetate 90, KCl 20, MgCl_2_ 3, CaCl_2_ 1, EGTA 3, HEPES 40 and NaOH ~20 to give a pH of 7.2. For voltage-clamp experiments, the same solutions were used (estimated junction potential was 9 mV; not corrected). In experiments with symmetrical potassium concentrations, this extracellular solution was similar except for NaCl 30 mM and KCl 110 mM (estimated junction potential was 6.2 mV; not corrected) [16, 17].

Membrane conductance (*G*) was estimated in voltage-clamp experiments (holding potential = -30 mV) by the application of negative 15 mV voltage steps of 50 ms at 0.5 Hz. After measuring the current (*I*) obtained during these steps, *G* was calculated based on Ohm´s Law, where *G* = 1/*R* and *R* = 15 mV/*I*. These conductance values were compared among conditions (control and drug-treated).

When specified, tetraethylammonium chloride (TEA, 15 mM), 4-aminopyridine (4-AP, 2 mM), cesium chloride (CsCl, 1 mM), cadmium chloride (CdCl_2_, 100 μM) and TTX (0.5 μM), were added to the extracellular solution in order to block voltage-dependent potassium, cationic, calcium and sodium currents. This combination of drugs constituted the cocktail solution. In some experiments, this cocktail was supplemented with apamin (200 nM), paxilline (1 μM), clemizole (10 μM) and ruthenium red (10 μM) in order to bock, respectively, calcium-dependent potassium channels (SK and BK), TRP channels (TRPC5, TRPC4, TRPC3 and TRPC6) and some K2P channels (TRAAK, TREK-2 and TASK3), and it was called cocktail B. All solutions used were kept between 290 and 300 mOsm. In some experiments, we pre-incubated our primary cultures with 1 μM spadin during 1.5 hour (from a 1 mM stock in water).

TEA, 4-AP, CsCl, CdCl_2_, capsaicin and fluoxetine were acquired from Sigma-Aldrich (Madrid, Spain). TTX, clemizole hydrochloride, apamin, paxilline, ruthenium red, spadin and BL-1249 were acquired from Tocris Bioscience (Abingdon, United Kingdom). ML67-33 was acquired from Aobious (Gloucester, Massachusetts). Riluzole was acquired from HelloBio (Bristol, United Kingdom). All drugs were dissolved in water except for riluzole (DMSO stocks of 300 mM and 1000 mM), ML67-33 (DMSO stock of 50 mM), BL-1249 (DMSO stock of 50 mM), paxilline (DMSO stock of 2 mM), fluoxetine (DMSO stock of 100 mM) and capsaicin (ethanol stock of 3 mM). Final DMSO and ethanol concentrations ranged 0.1–0.2%, which has been tested not to affect cell physiology.

Sampling frequency was 2 kHz (filtered at 0.5 kHz) for voltage-clamp and 10 kHz (filtered at 5 kHz) for current-clamp experiments. Data were digitized using a Digidata 1440A and analyzed offline using the software pClamp10 (Molecular Devices, Union City, USA). Plotting and statistical analysis were made using the software Origin 7.5 (OriginLab Corporation, Northampton, USA). Averages represent mean±SEM and statistical differences were assessed using two sample Student’s t-tests or ANOVA. A Shapiro-Wilk test was used to assess normality.

### Molecular biology, cell culture and transfection of tsA201 cells

Human TRESK (KCNK18) complementary DNA (cDNA) was cloned into pcDNA3.1 vector (Invitrogen, Carlsbad, CA). tsA201 cells, which are modified human embryonic kidney 293 cells, were transfected with the SV40 large T antigen (ECACC; Sigma-Aldrich, Gillingham, Dorset, UK). tsA201 cells were grown in a monolayer tissue culture flask maintained in growth medium that was composed of 88% minimum essential media with Earle’s salts, 2 mM L-Glutamine, 10% heat-inactivated foetal bovine serum, 1% penicillin (10,000 units.mL^-1^) and streptomycin (10 mg.mL^-1^), and 1% nonessential amino acids (Sigma-Aldrich, Pan Biotech, Fisher Scientific). The cells were stored in an incubator at 37°C with a humidified atmosphere of 95% O_2_ and 5% CO_2_. When the cells reached 80% confluency, they were split and resuspended in a four-well plate containing 13-mm diameter glass coverslips coated with poly-D-lysine (1 mg.mL^-1^) at a concentration of 7 x 10^4^ cells in 0.5 mL media, ready for transfection the following day.

Cells were transiently transfected using a modified calcium-phosphate protocol. 500 ng of pcDNA3.1 vector (Invitrogen) encoding human TRESK and 500 ng cDNA encoding for green fluorescent protein (GFP) was added into each well. The cells were incubated for 6–8 hours at 37°C in 95% O_2_ and 5% CO_2_. Following incubation cells were washed twice with a 1X phosphate-buffered saline solution (PBS), and incubated in 0.5 mL of fresh growth media overnight. The cells were used for electrophysiological recordings the following day.

### Whole-cell recording from tsA201 cells expressing TRESK channels

Currents were recorded from tsA201 cells transiently transfected with TRESK channels using whole-cell patch-clamp in a voltage-clamp configuration. A coverslip with transfected tsA201 cells was transferred into a recording chamber filled with an external solution composed of (mM): NaCl 145, KCl 2.5, MgCl_2_ 3, CaCl_2_ 1 and HEPES 10 (pH to either 7.4 or 8.4, using NaOH), mounted under an inverted microscope (Nikon Diaphot) with epifluorescence. External solution and modulatory compounds were superfused at a rate of 4–5 mL.min^-1^ at 20–24°C (room temperature). Complete exchange of the bath solution occurred within 100–120 seconds. Only cells that were transfected with GFP were selected for electrophysiological recordings. Patch pipettes were pulled from thin walled borosilicate glass (GC150TF, Harvard Apparatus, Edenbridge, UK) and had resistances of 3–6 MΩ when filled with pipette solution. The pipette solution contained (mM): KCl 150, MgCl_2_, 3; EGTA 5 and HEPES 10 (pH adjusted to 7.4 with KOH). Whole–cell currents were evoked using a “step-ramp” protocol. From a holding potential of -60 mV, cells were hyperpolarized to -80 mV for 100 milliseconds (ms) and then subjected to a step to -40 mV for 500 ms, then a step to -120 mV for 100 ms. This was followed by a 500-ms voltage ramp to +20 mV and a step back to -80 mV for another 100 ms before returning to the holding potential of -60 mV. This protocol was composed of sweeps lasting 1.5 seconds (s), including sampling at the holding voltage and was repeated once every 5 s. Currents were recorded using an Axopatch 1D patch clamp amplifier (Molecular Devices, Sunnyvale, CA) and analyzed using pCLAMP 10.2 software (Molecular Devices), Microsoft Excel (Redmond, WA) and GraphPad Prism 6 software (San Diego, CA). For analysis of outward current, we measured the current amplitude at -40 mV. Data are expressed as the mean ± 95% Confidence Intervals (CI), and *n* represents the number of individual cells (8) recorded on three separate days. Riluzole (Sigma-Aldrich) was made up in DMSO at a stock concentration of 10 mM.

## Results

We have previously shown that mouse NG neurons express riluzole-activated TREK channels using single channel recording and real time and conventional RT-PCR [6]. In the present study, we developed a novel way of classifying mouse nodose neurons in culture. This enabled us to investigate the responsiveness of the different subgroups of neurons to riluzole by conducting the whole-cell patch-clamp experiments.

### Main properties of NG neurons

**Fig 1:**
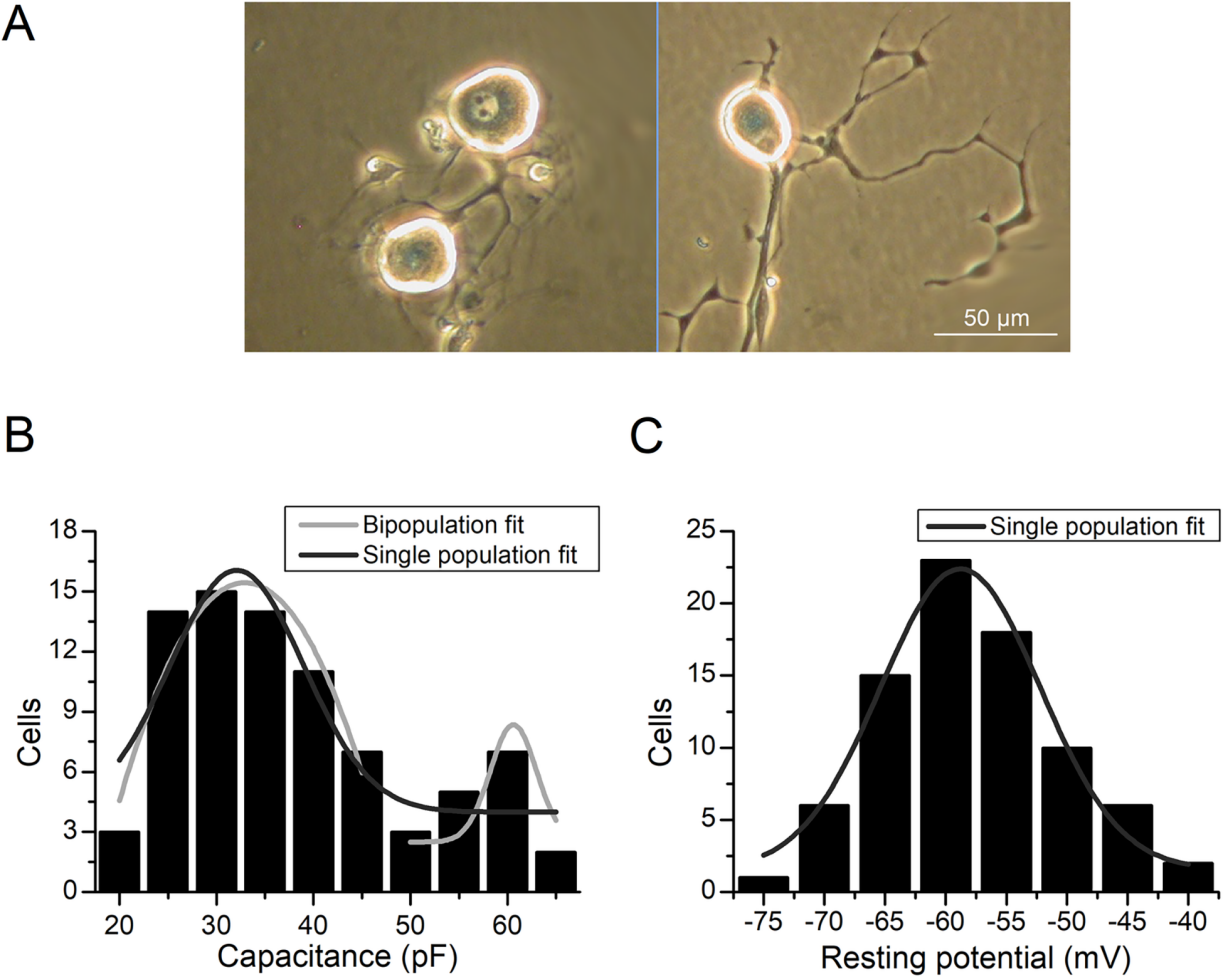
Basic properties of mNG neurons in culture. **(A)** Different micrographs (after 24 h. in culture) were taken with an inverted phase contrast microscope. The brightness of the membrane contour serves as indication of cell viability to perform patch-clamp experiments. **(B)** Frequency distribution of capacitance values for the experimental sample. Shapiro-Wilk test fails to detect a normal distribution (*P*<0.001, n = 81). **(C)** Frequency distribution of resting membrane potential values for the experimental sample. Shapiro-Wilk test reports a Gaussian distribution (*P* = 0.57, n = 81).

Mean capacitance of the isolated neurons was 34.8±1.4 pF (n = 81), which was very similar to that previously reported in rat isolated nodose neurons [11]. Although obvious morphological differences were not detectable by eye (Fig 1A), distribution of capacitance values failed to pass a normality test (Shapiro-Wilk test, *P*<0.001), suggesting the presence of at least two subpopulations of neurons with different cell body size (Fig 1B). Capacitance differences between A and C type cells were previously reported in NG neurons from rabbit [18], and two populations with different soma size have been reported in rat DRG neurons [10]. On the contrary, resting membrane potential values in the same experimental sample showed a clear Gaussian distribution (Shapiro-Wilk test, *P* = 0.57), suggesting no differences for this parameter among NG neurons (Fig 1C). Resting membrane potential values (-61.1±0.8 mV, n = 81) were similar to those previously described in cultured rat neurons [11, 19].

### Response to riluzole

**Fig 2:**
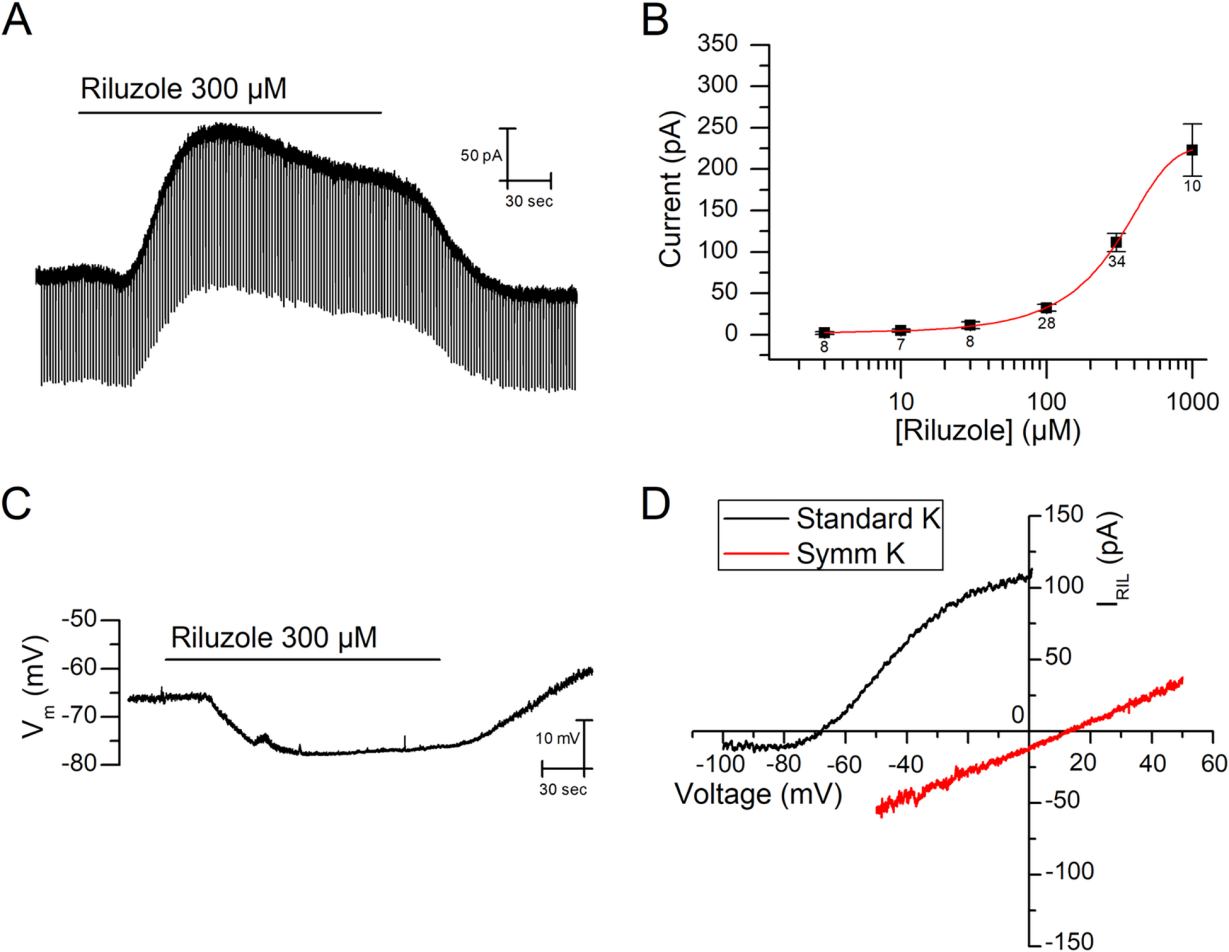
Riluzole-activated outward current and hyperpolarization. **(A)** Short (50 ms) voltage steps (to -45 mV) were applied at high frequency (50 Hz) to detect changes of membrane conductance in the presence of 300 μM riluzole and cocktail (holding potential -30 mV). **(B)** Dose-response curve representing the current activated by riluzole with the membrane clamped at -30 mV, in the presence of the cocktail. Estimated *EC*_50_ was 392.2±182.8 μM. Number of cells for each concentration are specified under the points. **(C)** Hyperpolarization induced by the application of riluzole, for 3 minutes, on the resting membrane potential (V_m_) in bridge mode-like experiments (*I* = 0). Note that this recording comes from a different cell than that depicted in A. **(D)** Current–voltage relationships for riluzole-induced currents in the presence of cocktail, obtained in response to negatively progressing voltage ramps. The current obtained in the control was subtracted from that obtained in the presence of 300 μM riluzole. Note that the strong outward rectification obtained in standard solutions (*E*_K_ = -90.7 mV) disappeared when symmetrical concentrations of potassium were used (*E*_K_ = 0 mV). Recordings were acquired from two different cells.

Most mNG neurons when clamped at -30 mV responded to 300 μM riluzole with an outward current of 86.6±8.6 pA (n = 56 of 60, Fig 2A), in the presence of the cocktail solution (see Material and Methods). Using small negative voltage steps (-15 mV, 50 ms at 0.5 Hz), we show that riluzole induced an increase in conductance of 1.51±0.39 nS (n = 8, *P*<0.01, Student's t-test) (Fig 2A). This indicates that the riluzole-activated outward current (*I*_Ril_) can only be attributed to the opening of ion channels rather than to the putative inhibition of sodium or calcium currents. The effect of riluzole was concentration-dependent (Fig 2B), reaching 222.8±31.5 pA at 1 mM (n = 10), with an extrapolated *EC*_50_ of 392.2±182.8 μM. In more than half of cells (33 of 56) the outward current activated by riluzole was clearly transient, as expected for heterologously expressed TREK-1 and TREK-2 currents [20, 21] and native *I*_Ril_ [7, 22].

Bridge mode-like experiments with the neurons at rest (*I* = 0) showed that riluzole (300 μM) hyperpolarized the resting membrane potential (V_m_) of mNG neurons by about 10 mV (-11.7±2.0 mV, n = 4, *P*<0.05, Student's t-test, Fig 2C). In 2 of these 4 cells, the riluzole-induced hyperpolarization was again transient, in agreement with the transient nature of *I*_Ril_. These results mimicked those reported for sympathetic neurons [7].

We wanted to confirm that the outward current activated by riluzole is a potassium conductance. We generated repetitive negatively progressing voltage ramps (100 mV/s, every 10 s) to investigate the inversion potential of *I*_Ril_ in standard conditions of potassium (equilibrium potential for potassium, *E*_K_ = -90.7 mV) and in symmetrical potassium (*E*_K_ = 0 mV). The current evoked by the ramp at the peak of the riluzole effect was subtracted from that obtained before riluzole was applied to define *I*_Ril_. In the presence of symmetrical potassium concentrations, *I*_Ril_ inversion potential shifted 87.8±4.1 mV (n = 7, *P*<0.05, Student's t-test) to values near zero (Fig 2D), very similar to the expected difference of *E*_K_ for both conditions (90 mV), and suggesting that this is indeed a potassium conductance. Furthermore, the I–V for *I*_Ril_ strongly rectified in standard but not at symmetrical potassium concentrations (Fig 2D). Note that in experiments using voltage ramps, 4-AP was added to the extracellular solution to prevent the activation of the transient outward K-current (*I*_K(A)_) during the step to depolarized potentials at the beginning of each ramp.

**Fig 3:**
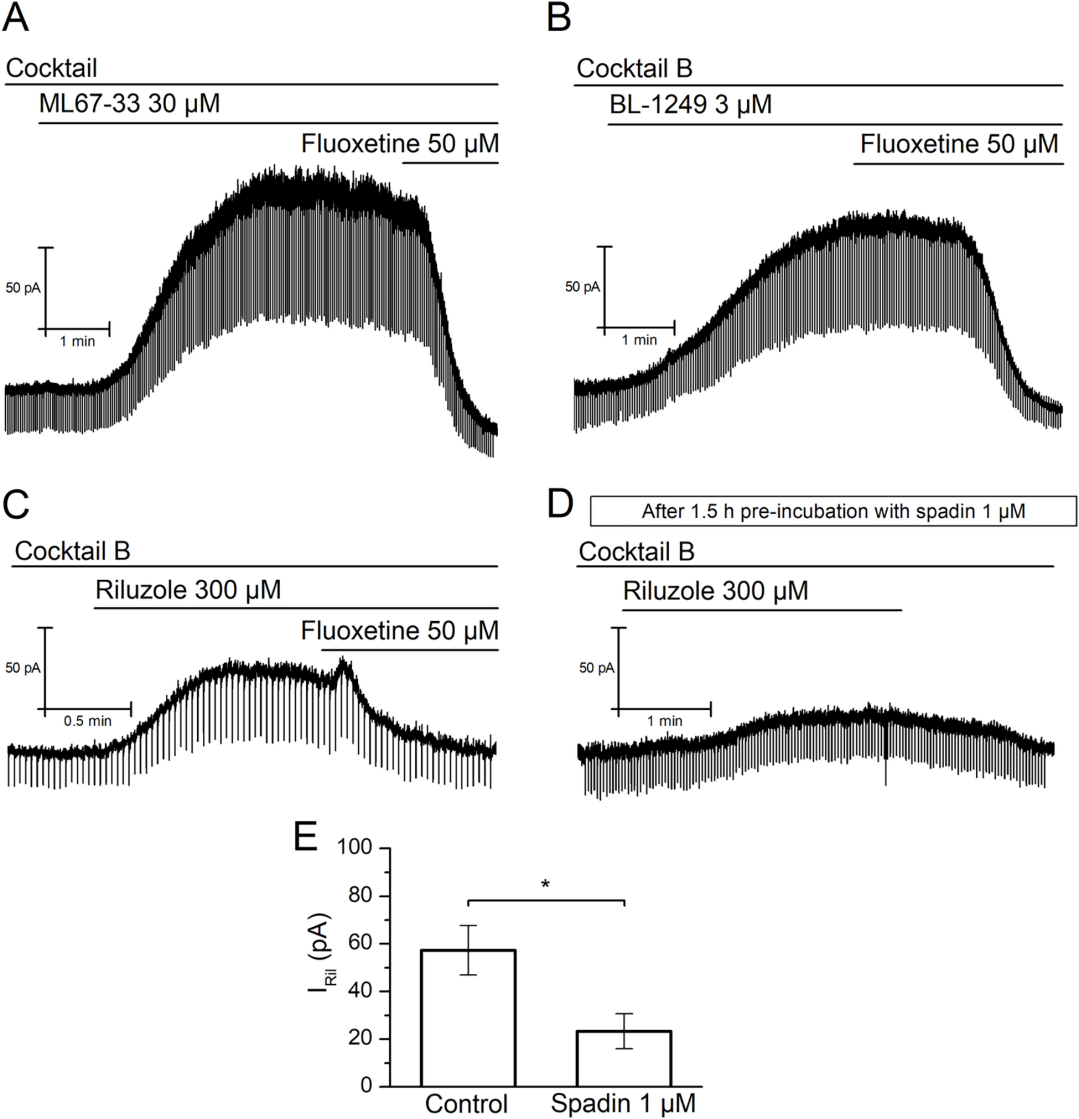
Riluzole-activated outward current is mediated by TREK channels. **(A)** Short (50 ms) voltage steps (to -45 mV) were applied at high frequency (50 Hz) to detect changes of membrane conductance in the presence of 30 μM ML67-33 and the cocktail solution (holding potential -30 mV). Note the clear inhibition of the *I*_ML_ by fluoxetine. **(B)** Short (50 ms) voltage steps (to -45 mV) were applied at high frequency (50 Hz) to detect changes of membrane conductance in the presence of 3 μM BL-1249 and cocktail B (holding potential -30 mV). Note the clear inhibition of the *I*_BL_ by fluoxetine. **(C)** Short (50 ms) voltage steps (to -45 mV) were applied at high frequency (50 Hz) to detect changes of membrane conductance in the presence of 300 μM Riluzole and cocktail B (holding potential -30 mV). Note the clear inhibition of the *I*_Ril_ by fluoxetine. **(D)** After 1.5 hour of incubation in 1 μM spadin, short (50 ms) voltage steps (to -45 mV) were applied at high frequency (50 Hz) to detect changes of membrane conductance in the presence of 300 μM riluzole and cocktail B (holding potential -30 mV). Note that the decrease of *I*_Ril_ in comparison to control conditions (C). **(E)** Summary bars describing the inhibitory effect that 1.5-hour incubation of the TREK-1 inhibitor spadin exert on the current-activated by riluzole.

Because riluzole is not a specific activator of the TREK subfamily channels, we wanted to confirm the effect of riluzole by testing a different compound, named ML67-33, reported to activate specifically the three members of the TREK subfamily but no other K2P channel [23]. Similarly to riluzole, when clamped at -30 mV most mNG neurons responded to 30 μM ML67-33 with an outward current of 139.6±25.8 pA (n = 24 of 28), in the presence of the cocktail solution. As previously, using small negative voltage steps (-15 mV, 50 ms at 0.5 Hz), we demonstrated that ML67-33 induced an increase in conductance of 2.04±0.47 nS (n = 7, *P*<0.001, Student's t-test) (Fig 3A), indicating that the ML67-33-activated outward current (*I*_ML_) is mediated by the opening of TREK channels.

Riluzole is a neuroprotective drug that in addition to TREK interacts with a variable number of channels [24]. Considering this, and also because the nodose ganglion is a complex ganglion composed by a heterogeneous group of cell types, we wanted to rule out the possibility that the effect of riluzole is mediated by other channels, including TRPC5, known to be activated by this compound [25]. To demonstrate that *I*_Ril_ is not mediated by other channels apart from TREK, we repeated our original experiment this time in the presence of a more supplemented cocktail of inhibitory drugs containing TEA, 4-AP, TTX, CdCl_2_, CsCl, apamin, paxilline and clemizole. Moreover, we added ruthenium red at a concentration known to block TRAAK, TREK-2 and TASK-3, but not TREK-1 channels [26–28] (cocktail B, see Methods). In the presence of cocktail B, riluzole still activated an outward current (57.3±10.4 pA, n = 10) (Fig 3C), which was comparable to that obtained when riluzole was applied in the presence of our original cocktail during interleaved experiments (54.2±13.6 pA, n = 14, *P*>0.05, Student's t-test). *I*_Ril_ was still accompanied by an increase in membrane conductance of 1.16±0.24 nS (n = 9, *P*<0.01, Student's t-test), indicative of the opening of channels.

Additionally, we wanted to further confirm our results obtained with ML67-33 and riluzole using a third compound, BL-1249, also described to activate TREK channels. Besides TREK-1, TREK-2 and TRAAK, BL-1249 activates TASK-3, therefore, we tested this compound also in the presence of cocktail B. Under these conditions, BL-1249 at a concentration (3 μM) described to mostly activate TREK channels [29, 30], induced an outward current of 97.0±24.4 pA (n = 9) (Fig 3B). During this BL-1249-activated outward current (*I*_BL_), membrane conductance increased significantly by 3.21±0.86 nS (n = 9, *P*<0.01, Student's t-test).

In order to demonstrate that these currents are indeed carried by TREK channels we applied fluoxetine, which inhibits TREK subfamily channels [31–33], at the peak of *I*_ML_, *I*_BL_ and *I*_Ril_. In the three cases, the activated currents were completely abolished by 50 μM fluoxetine (Fig 3A, 3B and 3C). Besides TREK-1, fluoxetine also inhibits TREK-2 and TRESK channels [32], therefore we tested if a more specific TREK-1 inhibitor could affect *I*_Ril_. Spadin is a synthetic partial peptide derived from the precursor form of the endogenous peptide of 44 aminoacids called sortilin. Spadin binds specifically to TREK-1 promoting its internalization [34–36]. We pre-incubated our primary cultures with 1 μM spadin during 1.5 hour and afterwards performed our patch-clamp recordings as usual. In the cells pre-treated with spadin, *I*_Ril_ in the presence of cocktail B was significantly reduced compared to control conditions to 23.4±7.3 pA (n = 11, *P*<0.05, Student's t-test) (Fig 3C, 3D and 3E). Noteworthy, in the spadin-treated cells, riluzole did not change membrane conductance significantly (0.44±0.20 nS, *P*>0.05, Student's t-test).

**Fig 4:**
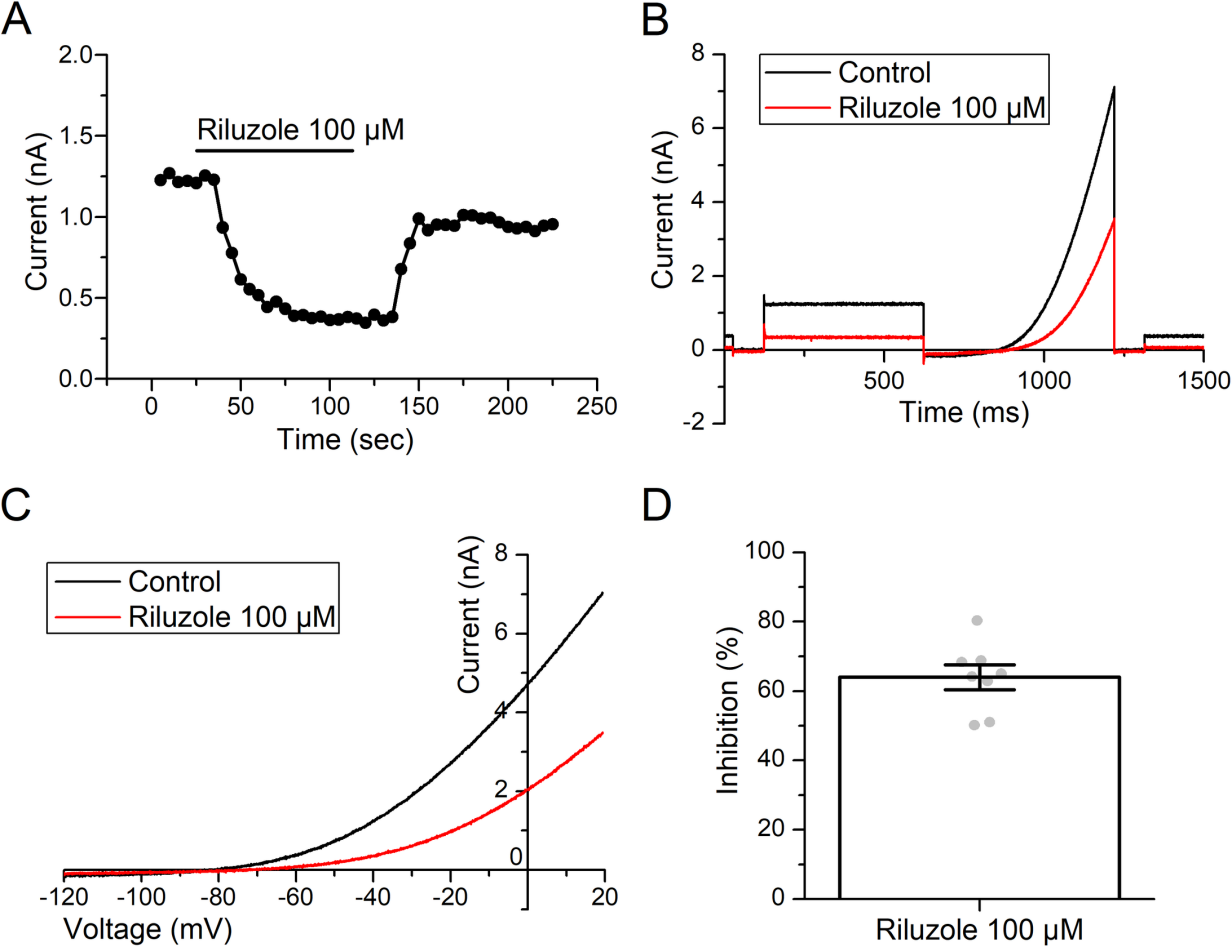
Riluzole (100 μM) is a potent inhibitor of human TRESK channels. **(A)** Bath application of riluzole (100 μM, black bar) rapidly and reversible inhibited TRESK current measured at -40 mV. **(B)** Representative traces of TRESK currents in a single cell evoked using the “step ramp” protocol described in the methods in the absence (black) and presence (red) of riluzole (100 μM). **(C)** Representative current-voltage relationships for TRESK channels in a single cell in the absence (black) and presence (red) of riluzole (100 μM). **(D)** Inhibition of TRESK channel current at -40 mV by riluzole (100 μM) in 8 individual cells from 3 different recording days (gray dots). Error bars represent the mean ± SEM.

Because TRESK is the most expressed K2P channel in mNG neurons [6] we wanted to discard that the outward current activated by riluzole is mediated by TRESK channels. For this purpose, we tested riluzole on a HEK293-derived cell line (tsA201) expressing TRESK channels. Strikingly, riluzole (100 μM) produced a substantive and reversible inhibition of human TRESK channels transiently expressed in tsA201 cells (Fig 4A). The current was inhibited over the entire voltage range recorded, from -120 to +20 mV (Fig 4B and 4C). At a holding potential of -40 mV, riluzole (100 μM) inhibited TRESK channel current by 64% (n = 8 cells from 3 days, 95% CI [56, 73], Fig 4D). This constitutes the first demonstration of the effect of riluzole on TRESK channels.

Taken together, these experiments indicate that the current activated by riluzole is mediated by TREK channels, and among them, TREK-1 appears to be the main mediator (see Discussion).

### Response to capsaicin

**Fig 5:**
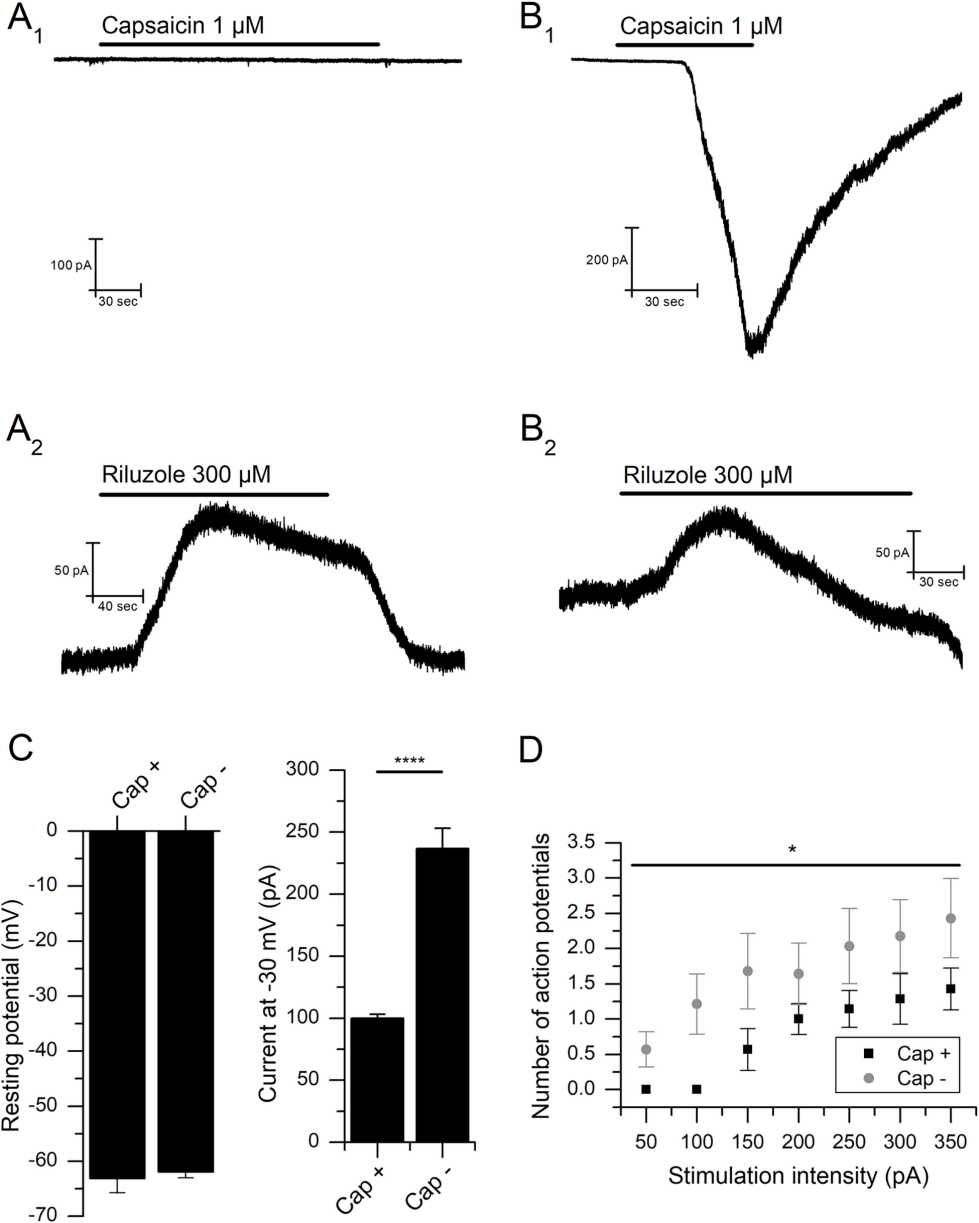
Effect of riluzole based on sensitivity to capsaicin. **(A)** Neuron unresponsive to capsaicin: **(A**_**1**_**)** Response to 1 μM capsaicin (V_m_ = -60 mV). **(A**_**2**_**)** Response of the same cell to 300 μM riluzole in the presence of the cocktail (V_m_ = -30 mV). **(B)** Neuron responsive to capsaicin: **(B**_**1**_**)** Response to 1 μM capsaicin (V_m_ = -60 mV). **(B**_**2**_**)** Response of the same cell to 300 μM riluzole in the presence of the cocktail (V_m_ = -30 mV). **(C)** Resting membrane potential levels are not dependent on capsaicin sensitivity (left). The steady-state outward current with the membrane clamped at -30 mV (*I*_-30_) is significantly higher in capsaicin-insensitive neurons (***P<0.001, right). **(D)** The number of action potentials in response to increasing one second current injections is smaller in capsaicin-insensitive neurons (*P<0.05).

In the present study, we tested the response of 68 mNG cells to capsaicin (1 μM) and 18 of them were responsive (Fig 5A1 and 5B1). In the activated cells, capsaicin induced a mean inward current of 1.02±0.22 nA (n = 18, Fig 5B1). Of the capsaicin-sensitive group, 8 of 9 neurons tested to riluzole responded with an outward current of 52.5±13.2 pA (n = 8, Fig 5B2). From the capsaicin-insensitive group 28 neurons were also treated with riluzole, 25 responded with an outward current of 72.4±10.2 pA at -30 mV (Fig 5A2), which was not significantly different from the capsaicin-sensitive group of neurons (*P*>0.05, Student's t-test).

When the membrane potential was clamped at -30 mV in control solution, without the presence of the blocker cocktail, an outward inactivating current (*I*_-30_) could be recorded in the different cell types. Interestingly, capsaicin-sensitive neurons showed an *I*_-30_ (100.0±13.2 pA, n = 16) that was significantly smaller than the *I*_-30_ of capsaicin-insensitive cells (236.8±16.3, n = 49, *P*<0.0001, Student's t-test) (Fig 5C right).

Resting membrane potential values were measured under current-clamp (*I* = 0 pA) conditions. No difference was observed between the capsaicin-sensitive (-63.1±2.6 mV, n = 15) and insensitive (-61.9±1.1 mV, n = 42) neurons (*P*>0.05, Student's t-test) (Fig 5C). However, the number of action potentials evoked after injection of increasing depolarizing current pulses was higher in capsaicin-insensitive cells compared to the sensitive ones (*P*<0.05, Two-way ANOVA) (Fig 5D).

### Response to TTX

Previously, the general assumption was that myelinated (A) nodose afferents express mainly TTX-sensitive Na_v_1.7 channels while only unmyelinated (C) nodose cells express a TTX-resistant Na_v_1.8 current [37–39]. However, recent experiments in isolated nodose neurons from adult rats and guinea pigs strongly indicate that TTX-resistant sodium currents are also expressed within the A group of fibers [11, 40].

**Fig 6:**
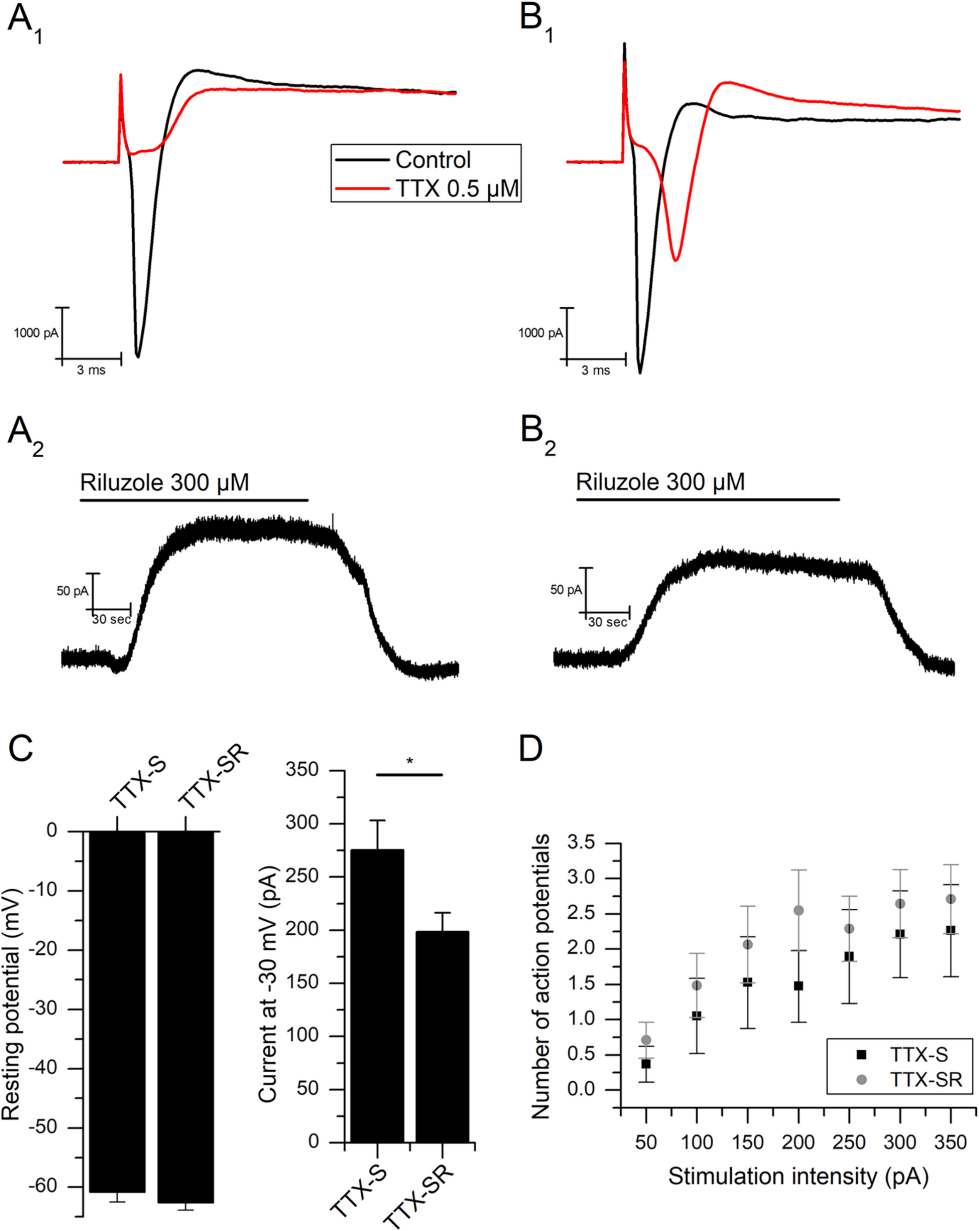
Effect of riluzole based on sensitivity to TTX. **(A)** Neuron with only TTX-sensitive (TTX-S) currents: **(A**_**1**_**)** Voltage-dependent inward currents in the presence of TEA, 4-AP, CsCl, and CdCl_2_, evoked with a voltage step from -80 to 0 mV (300 ms), before and after the addition of 0.5 μM TTX. **(A**_**2**_**)** Response of the same cell to 300 μM riluzole in the presence of the cocktail (V_m_ = -30 mV). **(B)** Neuron with both TTX-S and TTX-resistant currents (TTX-SR): **(B**_**1**_**)** Voltage-dependent inward currents in the presence of TEA, 4-AP, CsCl, and CdCl_2_, evoked with a voltage step from -80 to 0 mV (300 ms) before and after the addition of 0.5 μM TTX). **(B**_**2**_**)** Response of the same cell to 300 μM riluzole in the presence of the cocktail (V_m_ = -30 mV). **(C)** Resting membrane potential levels are not dependent on TTX sensitivity (left). Constant outward current with the membrane clamped at -30 mV is significantly higher in TTX-S neurons (*P<0.05, right). **(D)** Action potential firing in response to increasing one-second current injections is not dependent on TTX sensitivity.

We used brief voltage-steps (300 ms, -80 to 0 mV) in the presence of the cocktail of blockers to determine whether recorded mNG neurons expressed TTX-sensitive, TTX-resistant or both currents. As reported before [41, 42], solutions used to investigate the behavior of nodose neurons are not adequate to measure voltage-sensitive sodium currents, mainly because blockers of other channels (e.g. K^+^ channels) cannot be added to the pipette and intracellular ion concentrations cannot be modified. Even so, it is informative to state that after testing TTX on the inward current of 75 mNG neurons (2.9±0.2 nA), 27 had only TTX-sensitive currents (TTX-S, with mean control sodium currents of 2.6±0.2 nA, Fig 6A1) and 48 had both TTX-S and TTX-resistant components (TTX-SR, with mean control sodium currents of 3.1±0.2 nA) (Fig 6B1). Note that the inward currents obtained in our experiments often depict different temporal kinetics after TTX application. This is likely because the voltage in these experiments might not be entirely clamped [43], yet we could make a qualitative differentiation among TTX-S and TTX-SR currents to classify our cell types.

19 of 20 TTX-S neurons tested to 300 μM riluzole responded with an outward current of 90.4±14.2 pA (n = 19) (Fig 6A2). This was similar for the TTX-SR neurons, which had a response of 82.3±11.1 pA (n = 22 of 25 tested) to riluzole (*P*>0.05, Student's t-test) (Fig 6B2). However, the *I*_-30_ was significantly higher in TTX-S (275.2±28.0 pA, n = 26) than in TTX-SR neurons (198.4±17.8 pA, n = 46, *P*<0.05, Student's t-test) (Fig 6C right).

There was no difference observed in the resting membrane potential between the TTX-sensitive and the TTX-resistant cells (TTX-S: -60.8±1.7 mV, n = 24; TTX-SR: -62.6±1.3 mV, n = 42; *P*>0.05, Student's t-test) (Fig 6C left). Excitability was likewise comparable between the two groups (*P*>0.05, Two-way ANOVA) (Fig 6D).

### Grouping and classifying mNG neurons

Sensory neurons are generally classified into two to four categories ranging from A- to C-type neurons, a classification originally based on the CV of their axons [10, 18, 44]. When CV cannot be measured, for example in isolated neurons in primary culture, other features have been used to ascribe sensory neurons to those categories, e.g., action potential kinetics, pharmacology, soma size, expression of specific ionic currents, etc. [11, 45, 46]. The accuracy of these classifications is questionable and contradictory among the different studies. Therefore, we decided to classify our sample using the combination of two qualitative and unmistakable characteristics, namely, sensitivity to capsaicin and presence of the TTX-resistant sodium current. We have chosen these parameters because they cannot be misleading (for a discussion see [11]).

**Fig 7:**
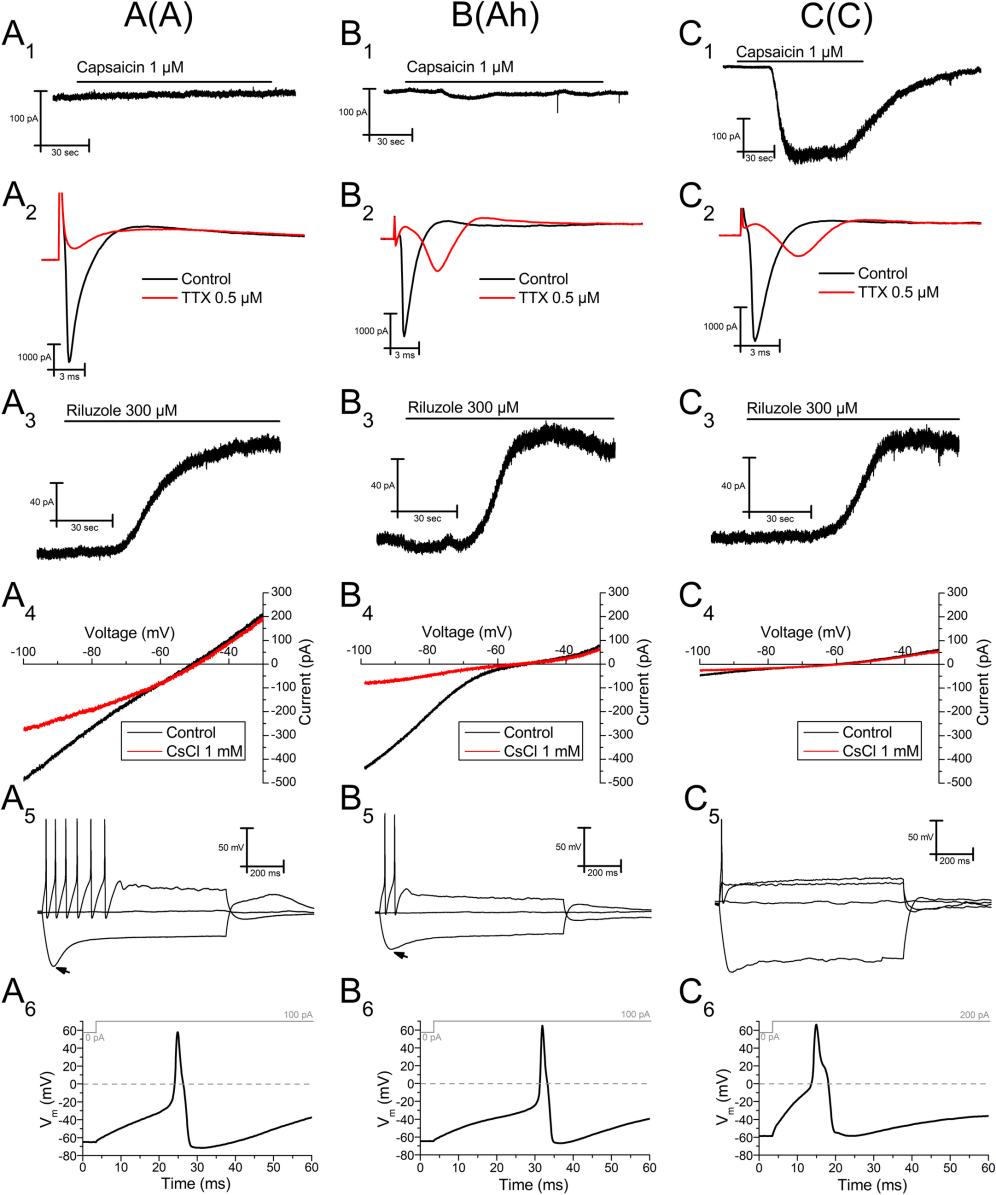
Characterization of three subpopulations of neurons in the mNG and response to riluzole. A-type **(A)**, Ah-type **(B)** and C-type **(C)** neurons are represented. **(1)** Only C-type mNG neurons respond to the application of 1 μM capsaicin (holding potential -60 mV). **(2)** Only A-type neurons show no TTX-resistant inward currents after a voltage step from -80 to 0 mV. **(3)** There are no significant differences in the outward current evoked by 300 μM riluzole among the different groups of neurons (holding potential -30 mV, in the presence of the cocktail). **(4)** Voltage ramps from -30 to -100 mV (10 mV/sec) were applied to study the magnitude of the h-current, revealed at hyperpolarized potentials after the application of 1 mM CsCl. All recordings in voltage-clamp experiments for each cell type are from the same neuron. **(5)** Changes in membrane potential after the injection of different current intensities (-100, 0, 100 and 200 (only C-type) pA) in the different groups of cells. Note the larger *sag* (arrow) seen after the hyperpolarizing pulse in A- and Ah-type cells, compared to C-type. Note that C-type neurons do not fire with current intensities under 200 pA. **(6)** Enlarged action potentials (first action potential in the depolarizing pulse in (5)) are represented to highlight their different duration among groups. Note that the measurement of the action potential duration is performed at half amplitude, where the characteristic hump of C-type cells is clearly distinguishable.

After testing the sensitivity of 62 mNG neurons to both capsaicin and TTX we split our sample into three categories (see Table 1). We believe this categorization may correspond to the classic ones *in vivo*. Following this assumption, we considered A neurons as those 21 cells that were irresponsive to capsaicin (CAP-, Fig 7A1) and had voltage-dependent sodium currents that were totally blocked by TTX (TTX-S, Fig 7A2). The 23 neurons that did not respond to capsaicin (CAP-, Fig 7B1) but had a clear TTX-resistant sodium current (TTX-SR, Fig 7B2) were defined as Ah cells. Finally, the remaining 18 neurons that responded to capsaicin (CAP+, Fig 7C1) and showed TTX-SR sodium currents (Fig 7C2) were considered C cells. Consistent with what has been previously reported in the somatosensory system [47], none of the TTX-S neurons responded to capsaicin (type A).

**Table 1:** Results obtained for different electrophysiological parameters in cells of the mNG.

	A-type	Ah-type	C-type
**TTX**	TTX-S	TTX-SR	TTX-SR
**Capsaicin**	No	No	Yes
**V**_**m**_ **(mV)**	-62.7±2.0 (18)	-61.1±1.7 (19)	-63.1±2.6 (15)
**Capacitance (pF)**	33±3.5 (21)	31.6±2.2 (23)	29.9±2.6 (18)
***I***_**-30**_ **(pA)**	244.1±28.8 (21)*	227.3±22.2 (23)†	120.0±23.5 (17)*†
***I***_**Na**_ **(nA)**	2.1±0.2 (18)	3.4±0.3 (20)	1.7±0.2 (14)
***I***_**h**_ **(pA)**	118.0±15.8 (15)*	81.5±13.5 (15)†	19.7±5.2 (11)*†
**Threshold (mV)**	-24.3±2.8 (9)	-24.8±2.5 (11)	-18.6±2.1 (7)
**AP**_**half-width**_ **(ms)**	2.2±0.2 (9)*	2.3±0.4 (12)	2.9±0.3 (7)*
**VD**_**max**_ **(mV.ms**^**-1**^**)**	117.4±20.7 (9)	148.4±13.7 (12)	122.1±9.4 (7)
**VR**_**max**_ **(mV.ms**^**-1**^**)**	-62.3±7.2 (9)	-76.9±6.2 (12)	-59.5±3.7 (7)
**Area (mV.ms)**	221.9±27.8 (9)*	295.7±42.0 (12)	337.9±25.8 (7)*
***I***_**Ril 300**_ μ_**M**_ **(pA)**	62.7±10.3 (14)	97.2±15.8 (10)	52.5±13.2 (8)

Values are given as mean±SEM. TTX: sensitivity of voltage-dependent sodium currents to TTX; Capsaicin: response with an inward current to the application of capsaicin 1 μM; V_m_: resting membrane potential; *I*_-30_: magnitude of the outward current at -30 mV; *I*_Na_: sodium current measured with a voltage step from -80 to 0 mV (300 ms); *I*_h_: cesium-sensitive current at -100 mV; AP_half-width_: action potential duration measured at half amplitude; Threshold: threshold for action potential firing; VD_max_: maximum rate of depolarization; VR_max_: maximum rate of repolarization; Area: area under the AP curve; *I*_Ril_: positive current evoked after the application of riluzole 300 μM.

* and † indicate significant differences between cell types to the designated characteristic (One-way ANOVA, *P*<0.05).

Using this classification, A cells showed a mean sodium inward current of 2.1±0.2 nA, (n = 18), Ah cells of 3.4±0.3 nA (n = 20) and C cells of 1.7±0.2 nA (n = 14). Additionally, Ah and C cells showed clear TTX-resistant sodium currents. Note that the exact magnitudes of sodium inward currents were not statistically compared as this parameter was only considered from a qualitative point of view.

14 out of 15 A-type cells that were tested to 300 μM riluzole responded with an outward current of 62.7±10.3 pA; 10 of 13 Ah cells responded with 97.2±15.8 pA; and 8 of 9 C cells responded with 52.5±13.2 pA (*P*>0.05, One-way ANOVA) (Fig 7(3); see Table 1). These results indicate that all the cellular subtypes from the mNG equally express TREK channels.

### Cell type and hyperpolarization-activated current

Previous investigations found a correlation between the cell type and the amount of hyperpolarization-activated cationic current (*I*_h_) or the size of the voltage "sag" induced by negative current injections in the nodose ganglion [19, 48, 49]. We used a voltage-ramp (from -30 to -100 mV) which allowed us to measure *I*_h_ as the cesium-sensitive current at -100 mV. Using the same protocol we were also able to measure the steady-state current at -30 mV (*I*_-30_). *I*_h_ measured in C-type neurons (19.7±5.2 pA, n = 11, Fig 7C4) was significantly smaller than that measured in Ah (81.5±13.5 pA, n = 15, *P*<0.01, One-way ANOVA) (Fig 7B4) and A (118.0±15.8, n = 15, *P*<0.0001, One-way ANOVA) (Fig 7A4) neurons (Table 1). Our results indicate that, all mouse cell types expressed *I*_h_, as previously described in cultured rat nodose neurons [19, 48], where a larger *I*_h_ current in A than in C cells is also reported. To our knowledge, we are the first to confirm *I*_h_ in mouse NG neurons.

In addition, *I*_-30_ in C cells (120.0±23.5 pA, n = 17) was significantly smaller than that obtained from Ah (227.3±22.2 pA, n = 23, *P*<0.05) and A (244.1±28.8 pA, n = 21, *P*<0.01, One-way ANOVA) cells (Table 1).

### Cell type and current-clamp properties of nodose neurons

Current-clamp characteristics of rat NG neurons, especially action potential properties, have previously been used as a classification system [11, 49, 50]. In order to investigate the voltage behavior of mNG we used bridge mode-like recordings (*I* = 0) and applied depolarizing and hyperpolarizing current steps. Using the classification mentioned above we found that the resting membrane potential was homogeneous among the groups, -62.7, -61.1 and -63.1 mV for A, Ah and C cells, respectively (*P*>0.05, One-way ANOVA) (Table 1).

In general, mNG neurons showed a strong spike frequency adaptation upon application of depolarizing current injections (see also [51]). Although no robust differences were observed among groups (see Fig 7(5)), it is worth noting that C cells needed stronger current injections to fire the first action potential, as these cells never fired with current injections below 150 pA (50 or 100 pA, *P*<0.001, Two-way ANOVA) (Table 2). As expected from data on *I*_h_ currents, well known to be responsible for the sag at these negative voltages [19, 52], all mNG neurons presented sags, however this response tended to be slower and less pronounced in C cells (see Fig 7(5)).

**Table 2:** Number of action potentials triggered after the injection of depolarizing current pulses.

Current injected (pA)	Total sample (n = 79)	A-type (n = 9)	Ah-type (n = 13)	C-type (n = 7)
**50**	0.77 ± 0.17	0.44 ± 0.44*	0.54 ± 0.27†	0.00*†
**100**	1.53 ± 0.30	1.67 ± 1.03*	1.00 ± 0.44†	0.00*†
**150**	1.90 ± 0.32	2.44 ± 1.26	1.54 ± 0.68	0.57 ± 0.30
**200**	2.18 ± 0.32	2.44 ± 0.93	1.46 ± 0.62	1.00 ± 0.22
**250**	2.25 ± 0.30	2.89 ± 1.30	1.54 ± 0.63	1.14 ± 0.26
**300**	2.44 ± 0.29	3.22 ± 1.13	1.69 ± 0.71	1.29 ± 0.36
**350**	2.61 ± 0.29	3.22 ± 1.22	1.92 ± 0.81	1.43 ± 0.30

Values are given as mean±SEM.

* and † indicate significant differences between groups (*P*<0.001, Two-way ANOVA).

Note that not all of the total sample of current-clamp recorded cells were classified.

Action potentials triggered in mNG neurons often showed a clear hump in the descending phase. This characteristic was always present in C cells and occasionally in Ah, but very rarely in A cells (only in one neuron), meaning the action potential duration was significantly wider in C (2.9±0.3 ms, n = 7, Fig 7C6) compared to A (2.2±0.2 ms, n = 9, Student´s t-test, *P*<0.05) (Fig 7A6) cells. The action potential duration of Ah cells (2.3±0.4 ms, n = 12, Fig 7B6) was not statistically different from the other two groups (Student´s t-test, *P*>0.05) (see Table 1). The action potential threshold was not significantly different among groups (A-cells -24.3±2.8 mV; Ah-cells -24.8±2.5 mV; C-cells (-18.6±2.1 mV; *P*>0.05) (Table 1).

## Discussion

The nodose ganglion contain the cell bodies that give rise to sensory endings of vagus nerves. It therefore represents the gateway for the fibers that convey sensory information from a number of targets, including the airways, heart, lungs, and stomach [8]. It is mainly an unconscious sensitivity resulting in the generation of homeostatic responses and reflexes. Considering the variety of stimuli that the NG sensitive endings detect, we hypothesized that K2P channels play an important role in the physiology of these neurons. Our data demonstrated the presence of riluzole-activated TREK currents in all three groups of nodose ganglion neurons. The activation of these currents induced membrane hyperpolarization.

### Mouse nodose ganglion neuronal subtypes

The NG is a complex structure containing distinct cell groups. Neurons with small cell bodies constitute unmyelinated, slow conducting C-fibers, and the largest cell bodies are the origin of fast-driving myelinated A-fibers. This traditional classification is based on the extensively studied dorsal root ganglion neurons, where cells are functionally classified due to fiber speed [10, 44, 53]. More recent studies consider three or four subpopulations of neurons within the NG by further subdivision of A or C cells into two subgroups [11, 40, 54, 55].

When recording from isolated cultured neurons, it is not possible to calculate fiber speed, thus other strategies, mainly electrophysiological properties and/or pharmacology, must be used. It is generally accepted that capsaicin sensitive neurons are C-type cells (giving rise to C-fibers) [40, 56]. However, C-fibers originating from the jugular-nodose ganglia and innervating the mouse lung can be further divided into two subtypes: a capsaicin and bradykinin sensitive group, which are very slow conducting (0.3–0.7 m.s^-1^), and a capsaicin and bradykinin insensitive group, which are slightly faster (0.7–1.5 m.s^-1^). Interestingly most cells (80 of 87) described in those studies were sensitive to mechanical stimuli, and 90% were activated by ATP [12, 57]. In addition, a large amount of mouse DRG neurons, which were supposedly unmyelinated C-fibers, were reported to be capsaicin-insensitive [58].

Due to this heterogeneity, we initially attempted to classify nodose neurons following similar parameters to those used by Li and Schild [11] in cultures of rat nodose neurons. Nevertheless, after analyzing action potential parameters (voltage threshold, rising time and descending time) in our sample of neurons, despite similar methodologies being used, we realized that the range of the values obtained were different from those reported by these authors. Furthermore, it has been demonstrated that although one-third of the mouse NG cells showed a distinct hump on the falling phase of the action potential, nearly all of them conducted in the C-fiber range, suggesting that the morphology of the action potential is not enough to classify nodose neurons [59]. Considering all this, we concluded that a pharmacological approach would provide us with more reliable information regarding the classification of the isolated mouse NG neurons *in vitro*.

In the present study, the application of TTX revealed two subpopulations, referred to as TTX-S and TTX-SR. These groups were defined by the blockade that this toxin exerted on sodium voltage-dependent currents during voltage-clamp recordings [19, 60]. Similarly, we also found that cells could be classified based on their sensitivity to the non-selective cationic TRPV1 channel agonist, capsaicin. Some cells showed a clear response to the application of capsaicin, but another separate group did not, confirming previous findings [5]. We then decided, in order to classify the cells, we would consider the complementary response to both compounds. We established the existence of three clearly differentiable and unmistakable subpopulations of NG neurons, namely, C-type (cap +, TTX-SR), Ah-type (cap-, TTX-SR) and A-type (cap-, TTX-S). Using this categorization, we could not detect major features in the cell morphology that would allow us to distinguish between the groups when seen under the phase-contrast microscope. That said, analysis of the capacitance values suggested the existence of at least two subpopulations of mNG neurons based on soma size, as previously described for other species [50, 61]. On the contrary, there were no differences observed in the resting membrane potential among the groups (average around -60 mV), this is consistent with previous data describing the resting membrane potential of NG neurons from other species [51, 62]. *I*_h_ was found to be larger in A-type cells compared to C-type, as described in rat NG neurons [19]. The sustained current measured at -30 mV was significantly smaller in C-type neurons compared to A- and Ah-type. This contrasts with the lower tendency of A-type neurons to adapt in response to depolarizing current pulses. Although *I*_-30_ has been ascribed to the presence of M-current in the rat NG neurons [51], we hypothesize that other leak conductances (e.g. K2P channels) might account for a significant component of the current measured at this potential.

Sensory fibers are classically divided into different subgroups (Aβ, Aδ and C) according to conduction velocity. As our classification criteria is based only on the pharmacology applied to isolated somas in a primary culture from the mNG, caution must be taken when the neuron types defined in this and other works [11] are to be compared with the classical classifications. We believed though, that several characteristics indicate that our proposed classification is functionally relevant. In particular, we hypothesize that the A-type neurons described in our study would correspond to the somas that give rise to Aβ-type fibers, described to have the largest diameter, largest h-currents and action potentials of short duration, and involved in the detection and rapid transmission of mechanosensitivity to tissue distension changes that take place in the upper airways [54]. C-type neurons, responsive to capsaicin and always presenting TTX-resistant sodium currents, would correlate to unmyelinated C-fibers, classically known as nociceptive receptors. These are slow-conducting fibers that respond to potentially noxious mechanical forces in a graded fashion [63]. Lastly, following the nomenclature given by Li and Schild [11], we named Ah those neurons that share characteristics with both A and C-type neurons. We conjecture that these cells might correspond to Aδ-fibers, classically referred as cough detectors [54]. Aδ-fibers are slower than Aβ-fibers and, like C-fibers, they express both TTX-sensitive and resistant sodium currents [11, 64].

In our study, C-type neurons appear to be the least abundant group of neurons within the mNG. This contradicts classical studies quantifying fiber types in rabbit and guinea pig NG [18, 62, 65], as well as more recent reports in mice [12, 59]. However, it is worth noting that none of these studies were performed in primary cultured isolated cells, thus the differences in the technique may account for such discrepancies. This is supported by the recent study showing a lower proportion of capsaicin-responsive cells recorded from isolated cells of the mouse NG [66].

### Mechanosensation in the nodose ganglion

It has been suggested that the capsaicin-sensitive TRPV1 receptor might mediate, directly or indirectly, the transduction of mechanical stimuli [67–69]. However, only a quarter of gastric mechanosensitive neurons from mouse nodose ganglion are immunoreactive for TRPV1 [59]. Additionally, Berthoud et al. [70] reported that approximately only a third of vagal afferents that innervated the stomach respond to mucosal application of capsaicin. Similarly, mechanosensitivity of bronchopulmonary C-fibers remained unaffected in TRPV1-/- mice [57]. This indicates that other groups of mechanosensitive receptors/channels, in which TREK channels are included, may also play a relevant role in sensorial transduction [6]. Channels of the TREK subfamily (TREK-1, TREK-2 and TRAAK) are particularly mechanosensitive [4]. Accordingly, TREK-1 is abundantly expressed in organs and tissues in which mechanosensitivity is relevant, such as lung, uterus, heart, skeletal muscle, stomach, intestine, colon or bladder [71]. In contrast, expression of TREK-2 channels is lower or even absent in these organs [21] (but see [72]), and TRAAK channels are mainly expressed in the central nervous system [73, 74]. Channels belonging to the TREK subfamily are expressed in DRG [75, 76] and trigeminal ganglion [77] sensory neurons, where they have an important role in the transmission of somatic mechanic information to the central nervous system. More specifically, TREK-1 expression in small and medium DRG neurons was confirmed using immunohistochemistry, where a lower expression was observed in larger sensory neurons [75]. It is therefore tempting to speculate that these channels could also be expressed in sensory neurons (visceral afferent neurons), carrying information about internal unconscious mechanical changes that are important for responses of the autonomic nervous system.

### On the nature of the riluzole–activated current

It is well known that both riluzole and fluoxetine can modulate several channel types [24, 32]. However, the present results and those published before strongly indicate that, in the nodose ganglion neurons, the riluzole-activated current is mainly generated by the activation of TREK-1 potassium cannels belonging to the TREK subfamily of the K2P family. The evidence is as follows:

1. Riluzole, ML67-33 and BL-1249, drugs known to activate potassium TREK channels, all induced an outward current at -30 mV accompanied of a conductance increase clearly indicating the opening of potassium channels rather than the closure of sodium, calcium or cationic channels. Consistently, when the concentration of extracellular potassium was altered, the change in the reversal potential of the current activated by riluzole closely followed the change in the equilibrium potential for potassium. Additionally, experiments addressing *I*_Ril_ and *I*_BL_ were conducted in a solution (cocktail B) that included blockers for sodium (including the persistent sodium current), calcium (N, L, P/Q and R) and cationic (including h, TRPC5, TRPC4, TRPC3 and TRPC6) voltage-activated channels (using TTX, Cd^++^, Cs^+^ and clemizole). It is worth noting that the activation of any of these channels should produce an inward current and not the outward current always seen when riluzole and the other two more specific activators of TREK currents are applied. These results demonstrate that the current activated by riluzole is mainly flowing through potassium channels.
2. In our experiments we also used blockers of non-K2P potassium channels to preclude the activation of voltage-dependent channels such as delayed rectifiers, A-type, M-type or inwardly rectifying potassium channels as well as calcium-activated potassium currents like SK and BK (using TEA, 4-AP, apamin and paxilline). Interestingly ML67-33, an activator of TREK currents but not affecting KCNQ2 (M) channels [23] still induced an outward current very similar to that evoked by riluzole. This pharmacological approach, and the fact that the current activated by riluzole is not rectifying in equimolar concentration of potassium, strongly indicates that it is carried through voltage-independent background K2P potassium channels.
3. To our knowledge, among K2P channels, only the TREK subfamily is activated by riluzole. Our laboratory has reported before that nodose ganglion neurons express several K2P channels, being the most expressed TRESK and TREK-1 when compared with the expression of other K2P channels. Hence, we tested the effect of riluzole in heterologously expressed TRESK currents and show that riluzole inhibits, rather than activates, TRESK currents. Therefore, the outward current induced by riluzole cannot be due to the activation of TRESK channels, although a reduction of the current by their inhibition cannot be ruled out with this experiment (but see below). Our cocktail B contained ruthenium red at a concentration that inhibits TASK-3 [26, 27]. On the other hand, TASK1, TASK-3 and TRESK have a very low sensitivity to fluoxetine when compared to *I*_Ril_ and TREK-1 currents [7, 32, 33, 78]. TWIK-1 should be blocked by TEA [1] and THIK-1 is inhibited by riluzole [79]. Finally, both BL-1249 and ML67-33 are known to activate TREK channels and they induce a current very similar to that induced by riluzole in nodose neurons [23, 30]. Importantly, ML67-33 strongly activates the three members of the TREK subfamily but not TASK-1, TASK-2, TASK-3 or TRESK [23]. All these factors and the fact that the outward current activated by riluzole was strongly reduced by spadin, a drug not affecting TASK-1 nor TRESK channels [36, 80] indicate that channels of the TREK subfamily should be mediating *I*_Ril_.
4. It is more difficult to discern which TREK channel subtype underlies *I*_Ril_. More than half of cells tested to riluzole responded with a transient current, which is characteristic of TREK-1 and TREK-2 because, unlike TRAAK, these channels are inhibited by the secondary increase of cAMP induced by riluzole [20, 21]. This transient effect of riluzole was also seen as a transient hyperpolarization in current-clamp experiments. However, it must be noted that the transient effect of riluzole has been originally reported in recombinant TREK-1 channels in COS cells by Duprat *et al*. [20], a very different scenario to the nodose ganglion cells under study here, where the riluzole-stimulated PKA pathway responsible for inhibition may operate very differently. On the other hand, distinguishing between TREK1 and TREK2 is particularly challenging because their properties are very similar. Still, several pieces of evidence allow us to reasonably argue that most of *I*_Ril_ flows through TREK-1 channels. We have recently demonstrated that TREK-1 channels are much more expressed than the other two members of this subfamily in these neurons [6]. Ruthenium red strongly inhibits TREK-2 and TRAAK but not TREK-1 channels at the concentration used [26] and did not affect the current activated by riluzole or BL-1249. Fluoxetine, a strong inhibitor of the riluzole-, ML67-33- and BL-1249-activated currents, also blocks TREK-1 with more potency than TREK-2, whilst TRAAK currents are essentially insensitive [31, 32]. Spadin has been reported to be a selective inhibitor of TREK-1 [34, 36] and in our hands it clearly reduced the current activated by riluzole. Finally, Tertyshnikova et al. used a similar approach to demonstrate that BL-1249 enhanced TREK-1 currents in human bladder cells [81].

### Physiological importance

It has been demonstrated that the inward, depolarizing h-current plays an important role in setting the resting membrane potential of rat nodose neurons, however the outward current that should balance the *I*_h_ at rest is unknown [19]. We therefore speculate that K2P channels may be responsible for the balancing outward current. We show that, in a similar manner to mSCG neurons [7], application of riluzole elicits a hyperpolarization on the resting potential of mNG neurons. This supports the evidence that riluzole has neuroprotective effects [24, 82], and suggests an important role of TREK channels in the modulation and establishment of the resting potential in viscerosensitive neurons of the mNG. Furthermore, the development of a clear classification of the mouse NG neurons allowed us to confirm that the distribution of TREK channels is independent of cell type, complementing previous reports describing the molecular expression of K2P channels in the nodose ganglion [5, 83]. Given that K2P channels are modulated by a variety of stimuli, e.g. mechanical stress, temperature or pH, future studies should focus on establishing whether the different stimuli produce different responses in the subpopulations of mNG neurons.
